# Retinal Layer Thickness and PET Centiloid Values in Cognitively Unimpaired Older Adults

**DOI:** 10.1002/alz70856_101202

**Published:** 2025-12-25

**Authors:** Ashley N. Price, Megan Stradtman, Savannah Doster, Jordan P. Sergio, Cyrus A. Raji, Mahsa Dolatshahi, Paul K. Commean, Mahshid Naghashzadeh, Abraham Z. Snyder, Sheng‐Kwei Victor Song, Tammie L.S. Benzinger, Matthew R Brier, Louisa I. Thompson, Alyssa N. De Vito, Peter J. Snyder, Jessica Alber

**Affiliations:** ^1^ University of Rhode Island, Kingston, RI, USA; ^2^ Butler Hospital Memory and Aging Program, Providence, RI, USA; ^3^ Alpert Medical School of Brown University, Providence, RI, USA; ^4^ Mallinckrodt Institute of Radiology, Washington University, St. Louis, MO, USA; ^5^ Mallinckrodt Institute of Radiology, Washington University in St. Louis, St. Louis, MO, USA; ^6^ Mallinckrodt Institute of Radiology, Washington University, School of Medicine, St. Louis, MO, USA; ^7^ Mallinckrodt Institute of Radiology, Washington University School of Medicine, St. Louis, MO, USA; ^8^ Washington University School of Medicine, St. Louis, MO, USA; ^9^ Butler Hospital, Providence, RI, USA; ^10^ Brown University Medical School, Providence, RI, USA

## Abstract

**Background:**

Beta amyloid (Aβ) PET results are quantified in centiloids to standardize cerebral Aβ burden, an established Alzheimer's disease (AD) biomarker. Spectral domain optical coherence tomography (SD‐OCT) imaging studies of the retina support retinal layer thinning as a biomarker of AD. Research examining the relationships between cerebral Aβ burden (in centiloids) and retinal layer thickness in preclinical AD remains understudied. This study aims to (1) examine the relationship between retinal layer thicknesses and PET centiloid values and (2) identify which retinal layers may be biomarkers of preclinical AD.

**Method:**

Heidelberg SPECTRALIS captured SD‐OCT images from 40 cognitively unimpaired older adults (ages 65‐80; mean=67.8). HEYEX software computed the ETDRS thickness maps for each retinal layer (mRNFL, GCL, IPL, INL, OPL, ONL, IRL, ORL, RPE). In this analysis, retinal structure measurements (thickness, volume) were averaged for each layer (i.e., full layer, central quadrant, inner ring, outer ring). PET scans using florbetaben were conducted within six weeks of retinal imaging. PET results were quantified via centiloid scale combined with visual reads to determine PET status: positive (*n* = 10) or negative (*n* = 30). Linear regressions, controlling for age, examined whether retinal thickness averages predicted centiloid values. Logistic regressions, controlling for age, examined whether retinal thicknesses predicted binary PET results.

**Result:**

Retinal mRNFL outer thickness significantly predicted PET centiloid value (*p* = 0.0206). Trends were seen in the relationship between mRNFL total layer thickness and PET centiloid (*p* = 0.0921) as well as mRNFL total layer volume and PET centiloid (*p* = 0.0679). The relationship between RPE outer thickness and PET status (*p* = 0.0757) and OPL total layer volume and PET status (*p* = 0.0572) was trending toward significance.

**Conclusion:**

The significant relationship between mRNFL and centiloid values aligned with previous work showing mRNFL thinning in MCI and AD. This is the first study to examine this relationship in a preclinical population. Retinal OPL findings replicate prior research by our group which demonstrated a relationship between OPL thickness and plasma ptau217, an indicator of cerebral amyloidosis. Further studies of longitudinal changes are needed to validate retinal biomarkers in preclinical AD.

